# Study on Refined Crushing Technology of RAP and Mechanical Properties of RAP-Doped Cement-Stabilised Macadam Base

**DOI:** 10.3390/ma18010147

**Published:** 2025-01-02

**Authors:** Peilin Liu, Bo Li, Fucheng Guo, Xu Wu, Tengfei Yao

**Affiliations:** 1 Gansu Industry Technology Center of Transportation Construction Materials Research and Application, Lanzhou Jiaotong University, Lanzhou 730070, China; peilinliu01@163.com (P.L.); libolzjtu@hotmail.com (B.L.); wuxuboshi@163.com (X.W.); tfyao@lzjtu.edu.cn (T.Y.); 2 Gansu Province Transportation Planning Survey & Designing Institute Co., Ltd., Lanzhou 730030, China

**Keywords:** reclaimed asphalt pavement (RAP), RAP-doped cement-stabilised aggregate, grade characteristics, agglomeration condition, mechanical properties, micro-morphology

## Abstract

In order to study the effect of the crushing process on the fine separation of reclaimed asphalt pavement (RAP) and the mechanical properties of cement-stabilised aggregate mixed with RAP, four crushing processes, namely small mesh hammer crushing, hammer crushing, jaw crushing, and double roller crushing, were used to separate the aggregate from asphalt in RAP materials. The effect of crushing on the grading characteristics and agglomeration condition of RAP material was investigated. RAP cement-stabilised aggregates were prepared and analysed for their mechanical properties and micro-morphology using RAP materials obtained from fine separation. The relationship between the RAP material properties and the mechanical properties of the RAP-added cement-stabilised aggregate was analysed on the basis of the tests. The results showed that crushing breaks down large-size RAP materials, leading to grade refinement, and that hammer crushing was the most effective in reducing the grade variability. The highest agglomerate dissociation rate of RAP material above 4.75 mm after small mesh hammer crushing treatment was 96.9%, and the residual mass ratios of RAP material in two grades of 0~3 mm and 3~5 mm after hammer crushing were lower than 90%. The unconfined compressive strength, splitting strength, and compressive resilience modulus of RAP cement-stabilised aggregate after crushing were greater than those of the uncrushed RAP cement-stabilised aggregate, and the crushing increased the amount of RAP in the mix to 60%. Compared with the unadulterated RAP cement-stabilised aggregate, the hydration products of the RAP cement-stabilised aggregate were reduced after crushing, and there were obvious gaps and discontinuities between the RAP material and the cement paste. The RAP gradation and agglomeration condition correlated strongly with the mechanical properties of the mixes, with RAP coarse aggregate agglomerates being the main cause of gradation variability. This paper provides theoretical support for the proposal of a pretreatment process to reduce the variability of RAP-doped cement-stabilised aggregate and improve the mechanical properties, and the research results are conducive to the recycling of high-volume RAP materials in the base.

## 1. Introduction

The number of RAP stockpiles increases rapidly as road maintenance mileage increases. The long-term storage of these RAP materials not only occupies a large amount of land resources and puts great pressure on the environment, but also causes huge economic losses such as dumping and maintenance [1,2,3]. Due to the serious agglomeration and large gradation variability in RAP materials, the utilisation rate of RAP materials in the recycling process of asphalt pavement is only 20~30%, which hinders the full use of RAP materials in road construction [4,5,6].

In order to reduce RAP material variability, break down agglomerates, and increase RAP material utilisation, researchers have separated aged asphalt and aggregates through a pretreatment process. Qiu et al. [7] developed a new RAP material fine separation technology based on the principle of impact crushing, and the pretreated coarse aggregate could be used directly as new aggregate. Martins et al. [8] investigated the effect of grinding on the binder properties and bitumen content of RAP materials by using grinding equipment to separate the oil from the stone. Li et al. [9] used a centrifugal crusher and multi-stage combined screening process to separate RAP materials, and the results showed that the crushing and screening process reduced the degree of agglomeration of RAP materials and improved the stability of gradation. Furthermore, the degree of agglomeration of the 5~10 mm and 10~15 mm RAP coarse aggregates obtained by pretreatment could be reduced to less than 21.6%. Ai et al. [10] quantitatively analysed the variability and properties of RAP materials during the rotational decomposition process, evaluated the laboratory performance of recycled asphalt mixtures with different percentages of RAP, and investigated the effect of RAP content. The results showed that rotary decomposition could result in recycled asphalt mixtures with a RAP content of more than 40%. Xu et al. [11] proposed a flexible and rigid combination of RAP material separation methods, compared the effect of this method with the factory separation method on the performance of RAP material and mixes, and analysed the dispersion uniformity of the RAP material in the mixes by techniques such as SEM and CT scanning. The laboratory method was effective in reducing 10~20 mm RAP stock agglomerates, ensuring a more homogeneous recycled mix, but reduced the dynamic stability of the mix. Currently, the research is focused on the pretreatment process on the separation effect of RAP material and the evaluation of the performance of the recycled mixture, the lack of different crushing methods on the RAP material gradation characteristics, and agglomeration condition performance comparative analysis. There has been no comparison on selecting the best crushing process that reduces agglomerates and the variability of RAP material.

The use of ordinary silicate cement as a binding material to stabilise the RAP material and apply it to the subgrade is a technique for reusing existing materials to achieve strength and stiffness. Thakur et al. [12] investigated the factors affecting the road performance of a cement-stabilised RAP base course using cyclic plate load tests and found that the replacement of natural aggregates with RAP material reduced the structural bearing capacity of the pavement base course. Ren et al. [13] and Kuchiishi et al. [14] investigated the unconfined compressive strength, splitting strength, and dynamic modulus tests of cement-stabilised RAP materials with different RAP material contents and found that the cement content as well as the RAP material content played important roles in the mechanical properties of cement-stabilised RAP materials, and recommended a material composition scheme for cement-stabilised RAP materials. Tian et al. [15] used overall blending and graded blending for the mix design, and their results showed that the use of natural aggregates with large particle sizes and recycled fine aggregates with small particle sizes had better overall performance. Based on the study of the asphalt film existing between RAP material and cement cementitious material, Huang et al. [16] found that the asphalt film had a buffering effect on the development of interfacial cracks, which enhanced the toughness of cement-stabilised RAP material. Farhan et al. [17] investigated the presence of weak interfacial transition zones between recycled aggregates and cementitious materials, where the presence of interfacial transition zones affected the flexural tensile strength of the cement-stabilised RAP materials. In the current research, the old road milling recycled RAP material without pretreatment directly into the mixture, and for cement-stabilised aggregates, the experimental research has achieved some results [18,19,20]. However, there is insufficient research on the enhancement effect of the crushing process on the performance of cement-stabilised RAP, the evaluation of the mechanical properties of cement-stabilised aggregates with RAP after crushing, and the micro-mechanism. Furthermore, there is a lack of research on the relationship between the properties of RAP materials and the mechanical properties of cement-stabilised aggregates with RAP.

**Figure 1:**
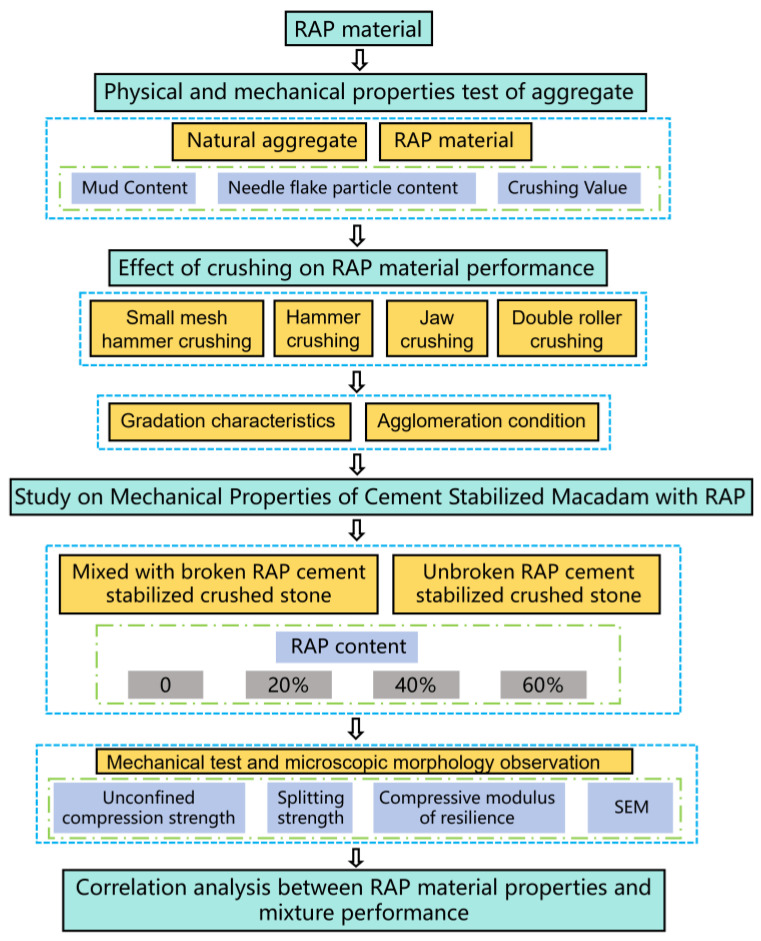
Technology roadmap.

In summary, in order to analyse the effect of different pretreatment processes on the fine separation of RAP materials and to study the mechanical properties of RAP-doped cement-stabilised aggregate after fine separation, this study adopted different crushing methods to deal with RAP materials and determined the grading characteristics before and after crushing. The effect of crushing on the dissociation of RAP material agglomerates in relation to the residual mass ratio was analysed using extractive sieving tests. The mechanical properties of the RAP-doped cement-stabilised aggregate after fine separation were investigated, and the micro-morphology of the mix was observed using SEM. The relationship between the RAP material properties and mechanical properties of the recycled mixes was analysed based on the test results. This study provides theoretical support for proposing measures to effectively reduce the degree of agglomeration of RAP materials, improve the pretreatment measures of gradation variability, and increase the utilisation rate of RAP materials in road base. The technology roadmap of this study is shown in Figure 1.

## 2. Materials and Methods

### 2.1. Materials

The RAP material used in the test came from the Highway Maintenance Centre in Baiyin City, Gansu Province, and was collected by milling the surface layer of the Kujie section of the G312 line. This section is a 57.23 km long dual carriageway motorway constructed in 2010. The original pavement structure consisted of a 4 cm AC-13 upper layer and a 5 cm AC-20 lower layer. In order to make the RAP material representative, 10 equally spaced points were selected from the top, middle, and bottom positions of the pile, and 30 kg of sample was taken from each point. The natural aggregate was a limestone produced by the Gansu Xinxinyuan Stone Factory (Baiyin, China). The physical and mechanical properties of the aggregates were tested according to the test methods in the specification, and the results are shown in Table 1, Table 2 and Table 3 [21]. The cement was a silicate cement produced by Gansu Qilianshan Cement Group Co., Ltd. (Lanzhou, China), and the indicators of setting time are shown in Table 4. The aggregate and cement properties all met the requirements of JTG/T F20-2015 [22].

### 2.2. RAP Material Crushing Process

**Figure 2:**
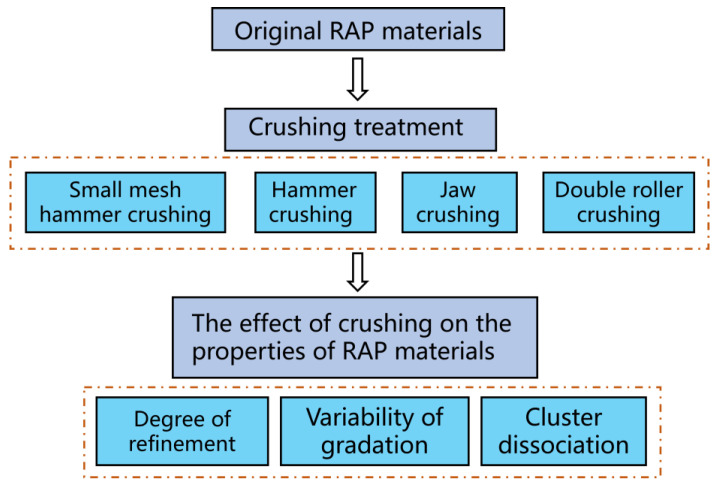
Crushing process flowchart.

The separation method of RAP material can be inspired from the stone crushing separation method. However, RAP materials have significant differences in the properties between natural aggregates and RAP materials due to the presence of an aged bitumen film on the aggregate surface. The small mesh hammer crushing and hammer crushing equipment used in this study was a hammer crusher produced by Henan Longyuan Machinery Co., Ltd. (Zhumadian, China). The screen size in the chamber of the hammer crusher is 4 cm, the small mesh hammer crushing screen size is 2.5 cm, and the rotor speed is 1300 rpm. Jaw crushing was carried out using an jaw crusher with a spindle speed of 850 rpm developed by Hebi Fengyuan Experimental Instrument Manufacturing Co., Ltd. (Hebi, China). The double roller crusher was produced by Shandong Huaji Heavy Industry Co., Ltd. (Weifang, China). The spindle speed of the crusher is 80 rpm. In this study, different crushing equipment were used to pretreat the RAP material in its original state, the mass of each group of RAP material sample was 5 kg, and the effect of stripping the surface of aged asphalt was achieved by using the abrasion, impact, and shear effect of the equipment on the RAP material. The research methodology used in this paper is shown in Figure 2.

The dried raw RAP material was poured into the inlet of the crusher, the equipment was started to crush the aggregate, and when it was discharged from the outlet, the separated aggregate and asphalt mortar were collected. The overall gradation of RAP material before and after crushing was determined using the sieve test, and five parallel tests were conducted for each crushing method to analyse the effect of the crushing process on the variability of the gradation of RAP material using the coefficient of variation of sieve passage rate. The coefficient of variation was calculated as shown in Equations (1)–(3):

(1) $$S=\sqrt{\frac{1}{n-1}\overset{n}{\underset{i=1}{\sum }}{\left(x_{i}-\bar{x}\right)}^{2}}$$

(2) $$\bar{x}=\frac{1}{n}\overset{n}{\underset{i=1}{\sum }}x_{i}$$

(3) $$COV=\frac{S}{\bar{x}}$$

where COV is the coefficient of variation of sieve throughput, dimensionless; S is the standard deviation of sieve throughput, and $\bar{x}$ is the mean value of sieve throughput.

In order to quantitatively analyse the changes in the degree of coarseness and fineness of the RAP material grades after crushing, the effect of different crushing processes on the refinement of the RAP material grades was investigated by using the fineness modulus, and the formula for calculating the fineness modulus is shown in Equation (4):

(4) $$u_{f}=\frac{\left[\left(\beta_{2}+\beta_{3}+\beta_{4}+\cdots \beta_{14}\right)-13\beta_{1}\right]}{\left(100-\beta_{1}\right)}$$

where $u_{f}$ is the modulus of fineness, %; $\beta_{1}$, $\beta_{2}$⋯$\beta_{14}$ are the cumulative sieve residue percentage of each level of sieve. The larger the modulus of fineness, the coarser the aggregate, and the smaller the aggregate, the finer the aggregate.

As the crushing process can strip the ageing asphalt on the aggregate surface, it achieves the purpose of breaking down the agglomerates. In order to compare the effect of different crushing processes to decompose the agglomeration of RAP materials, first, the sieving test was used to determine the gradation of RAP materials before and after the treatment of different crushing methods, and then the RAP materials were extracted and dried after the treatment of different crushing processes. The trichloroethylene during extraction removed the aged bitumen from the RAP material, and therefore the aggregate obtained after extraction was considered to be agglomerate free. The post-extraction aggregate gradation was then determined using a sieve test, as the closer the post-crushing gradation was to the post-extraction, the lower the agglomerate content of that sample was indicated. Therefore, this paper proposed the agglomeration dissociation rate index $R_{\mathrm{gd}}$ to evaluate the effect of crushing process on the agglomeration of RAP materials, where $R_{\mathrm{gd}}$ is the ratio of the graphical area enclosed by the gradation curves before and after crushing to the graphical area enclosed by the gradation curves after extraction and before crushing. The formula is shown in Equation (5):

(5) $$R_{\mathrm{gd}}=\frac{A_{c}}{A_{\mathrm{ec}}}$$

where $R_{\mathrm{gd}}$ is the agglomerate dissociation rate, %; $A_{c}$ is the area enclosed by the gradation curves before and after crushing; $A_{\mathrm{ec}}$ is the area enclosed by the gradation curves after extraction and before crushing.

### 2.3. Mechanical Properties Test of Cement-Stabilised Aggregate with RAP

**Figure 3:**
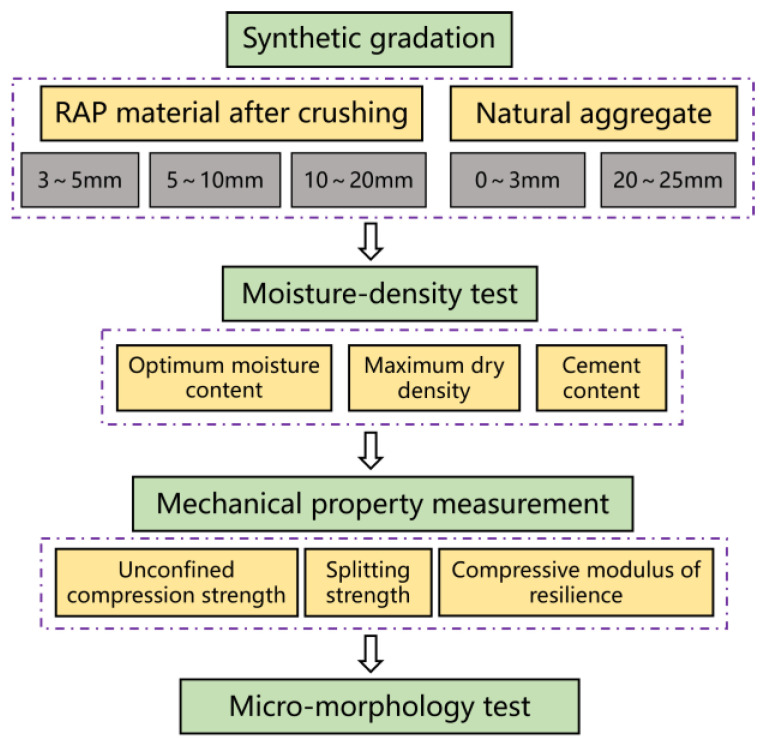
Flowchart for the mechanical property analysis.

The hammer-crushed RAP material and natural aggregates were left at room temperature for 24 h prior to the test to eliminate moisture, using 3~20 mm RAP material with 0~3 mm and 20~25 mm natural aggregates. The cement dosage was 2.5, 3.0, 3.5, and 4.0% and the RAP mixing rates were 0, 20, 40, and 60%. The optimum water content and maximum dry density were determined according to the Test Procedure for Inorganic Binder Stabilising Materials for Highway Engineering (JTG E51-2009) by a compaction test [23]. According to the results of the 7d strength test of mixes with different cement dosages and the strength requirement of the grass-roots level, the cement dosage was determined to be 2.5%. Cylindrical specimens with a diameter of 150 mm and a height of 150 mm were formed by hydrostatic pressing. Due to the poor adhesion of the RAP material to the cement paste, the mould was removed 0.5 h after the pressure was lifted, then the specimen was placed in a plastic bag and closed, before being moved to the recuperation room. After reaching the test age, the unconfined compressive strength, splitting strength, and compressive resilient modulus were determined for the crushed RAP cement-stabilised aggregates and the uncrushed RAP cement-stabilised aggregates. SEM was used to observe the micro-morphology of unbroken RAP cement-stabilised aggregates and 60% RAP cement-stabilised aggregates. The test flowchart is shown in Figure 3.

(1) Unconfined compressive strength

The specimens were cured until the sixth day into the water immersion, removed after 24 h, and measured for the unconfined compressive strength, calculated as shown in Equation (6):

(6) $$P=\frac{R_{c}}{A}$$

where $R_{c}$ is the unconfined compressive strength of the mixture, N; P is the maximum pressure at the time of destruction of the mixture, MPa; A is the cross-sectional area of the specimen, mm^2^.

(2) Splitting strength

Specimen maintenance to the specified age, then specimen was placed on the test machine and the loading rate controlled according to the deformation of 1 mm/min rate increase. The maximum pressure when the specimen was destroyed was recorded, according to Formula (7) to calculate the specimen splitting strength.

(7) $$P=\frac{2R_{i}}{\pi dh}(\sin2\alpha -\frac{a}{d})$$

where $R_{i}$ is the specimen splitting strength, N; P is the maximum pressure when the specimen destroys, MPa; d is the diameter of the specimen, mm; h is the height of the specimen, mm; α is the half-centre angle corresponding to the width of the compression strip, °; a is the width of the compression strip, mm.

(3) Compressive modulus of resilience

The calculated unit pressure on the specimen loading plate was 0.5~0.7 MPa, and the predetermined unit pressure was divided into 5–6 equal parts as the pressure value for each loading. The rebound deformation of the specimen was the micrometre reading at the time of loading minus the reading after unloading, and the rebound modulus was calculated in accordance with Equation (8):

(8) $$E_{c}=\frac{ph}{l}$$

where $E_{c}$ is the compressive resilience modulus, MPa; p is the unit pressure, MPa; h is the height of the specimen, mm; l is the specimen resilience deformation, mm.

### 2.4. Micro-Morphological Test of Mixes

The microscopic morphology of a material can reflect its macroscopic properties to a certain extent. The purpose of this study was to comprehensively compare and analyse the mechanical properties of RAP-added cement-stabilised aggregates with those of unadulterated RAP-added cement-stabilised aggregates to study the effect of RAP materials on the microscopic morphology of the cement-stabilised aggregates and to reveal the intrinsic connection between the macroscopic properties of mixes and microstructures as well as the micro-mechanisms of strength formation.

Therefore, in this study, scanning electron microscope (SEM) was used to observe the microscopic morphology of cement-stabilised aggregates without RAP and with 60% RAP. The same age specimens without the RAP cement-stabilised aggregate and with the RAP cement-stabilised aggregate were crushed and sampled separately. The length, width, and thickness of the test samples were 5 cm, 4 cm, and 2 cm, respectively. The samples were washed properly without damaging the surface texture and then dried. Since the sample itself was not a conductor, it was necessary to use conductive tape on the bottom and sides of the sample to connect it to the metal sample holder. The samples were placed in the ion sputterer along with the cradle to be coated to improve the conductivity of the samples, and the coating material was chosen to be Au film. An electron beam was emitted by the electron gun and the surface of the specimen was scanned using a raster-type progressive scanning method. Signals such as secondary electrons, backscattered electrons, and others were produced by the interactions between the electron beam and the sample atoms. These signals were collected and synchronised on a display device, resulting in a greyscale output that corresponded to the signal intensity line by line, thereby forming a microscopic image of the sample. SEM was used for comparative analysis by observing the hydration products of the samples and the microscopic morphology of the interfacial transition zone.

## 3. Results and Discussion

### 3.1. Influence of the Crushing Process on the Grade Characteristics of RAP Material

#### 3.1.1. Effect of Crushing on the Sieve Pass Rate of RAP Material

**Figure 4:**
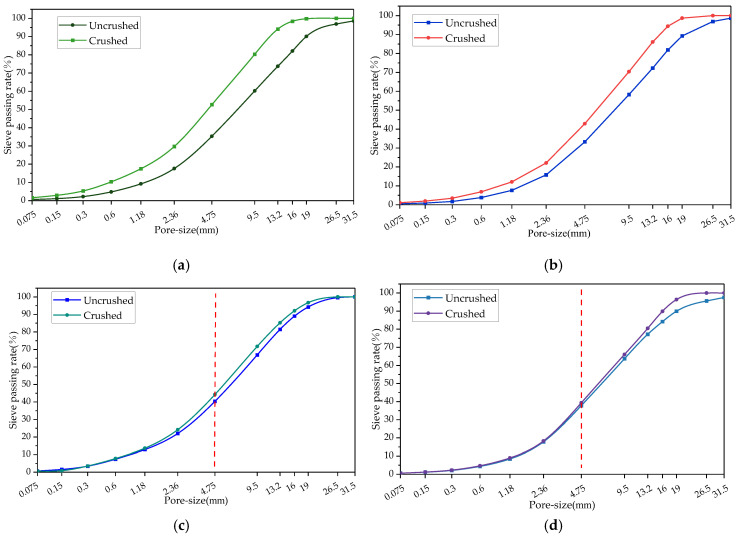
RAP gradation before and after the refinement process. (**a**) Small mesh hammer crushing. (**b**) Hammer crushing. (**c**) Double roller crushing. (**d**) Jaw crushing.

The effect of different crushing processes on the fine separation of RAP material was analysed using the sieve passing rate, and the grading curves of the RAP material before and after crushing are shown in Figure 4.

From Figure 4a,b, it can be seen that the passage rate of the full-size RAP material after crushing of the small mesh hammer type and hammer type was higher than that before crushing, and the grade curve after crushing with the small mesh deviated from the grade curve before crushing by a greater distance, which indicates that the RAP material after crushing of the small mesh hammer type is finer in particle size than that after crushing of the hammer type. It can also be seen from Figure 4a,b that the passages of 9.5, 13.2, and 16 mm pore sizes had the greatest degree of increase after treatment by both crushing methods compared with the pre-crushing period, which suggests that hammer crushing had the highest impact on particle refinement of these three particle sizes. From Figure 4c,d, it can be seen that the RAP material gradation curves after double roller and jaw crushing were above the pre-crushing curves, and the effect of the two crushing methods on the RAP fine aggregate gradation was smaller than that on the coarse aggregate, which indicates that particles larger than 4.75 mm can be more easily refined into small-sized particles after crushing [24].

#### 3.1.2. Effect of Crushing on the Fineness Modulus of RAP Material

**Figure 5:**
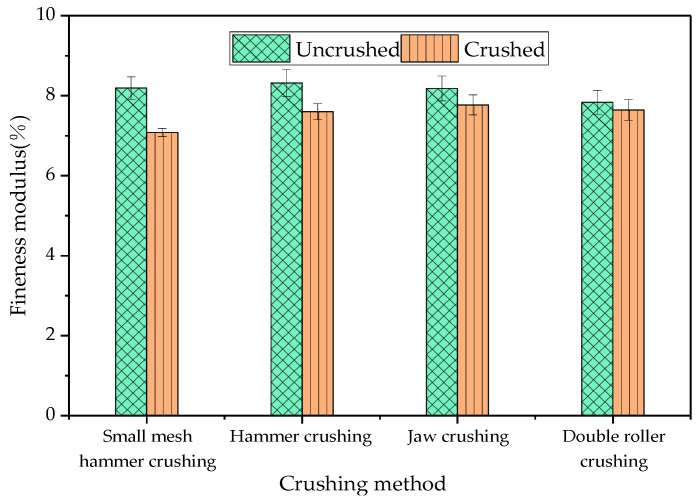
Effect of different crushing methods on the RAP fineness modulus.

According to the results of the gradation curves, it was found that the gradation of RAP material after the four crushing processes was finer than that before crushing. In order to quantitatively evaluate the influence of different crushing processes on the overall gradation characteristics of the RAP material, the fineness modulus index was used to analyse the degree of coarseness and fineness of the RAP material gradation. The results of the comparison of fineness modulus before and after the four crushing methods are shown in Figure 5.

**Figure 6:**
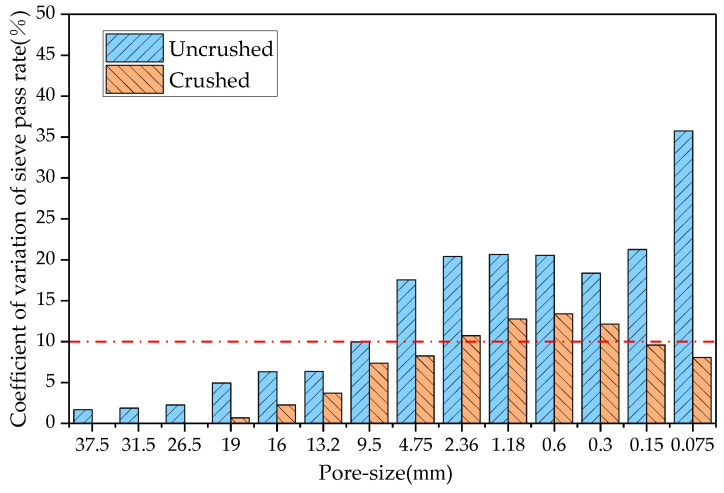
Comparison of the coefficient of variation of the sieve pass rate before and after hammer crushing.

Figure 5 shows the results of the comparison of the fineness modulus of the RAP material before and after the treatment of the four crushing methods. As can be seen from Figure 6, the fineness modulus of the RAP material after the treatment of different crushing methods was lower than that before the treatment. This was due to the fact that large size RAP material will decompose into small-sized aggregates under mechanical conditions, and the asphalt and dust on the surface of the pseudo-grains will be stripped by the equipment, leading to a reduction in the number of agglomerates, which results in the refinement of the RAP material gradation. It can also be found from Figure 5 that the RAP fineness modulus decreased by 13.55% after small mesh hammer crushing, the fineness modulus decreased by 8.65% after hammer crushing, the fineness modulus decreased by 5.01% after jaw crushing, and the fineness modulus decreased by 2.43% after double roller crushing, which indicates that small mesh hammer crushing had the most obvious effect on the grading refinement [25].

#### 3.1.3. Effect of Crushing on the Grade Variability of RAP Material

**Figure 7:**
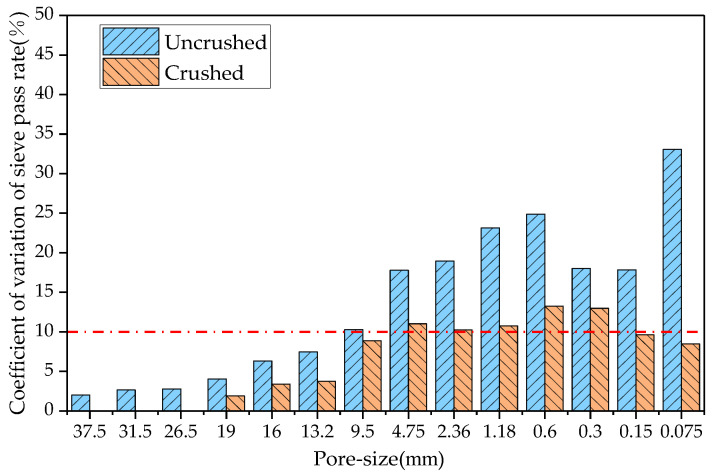
Comparison of the coefficient of variation of the sieve pass rate before and after small mesh hammer crushing.

**Figure 8:**
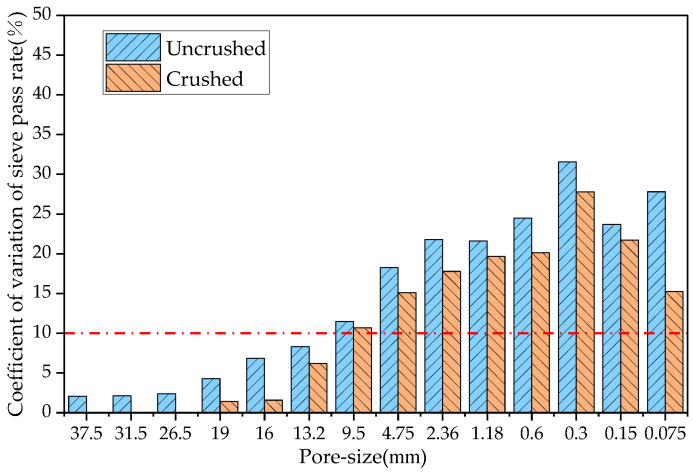
Comparison of the coefficient of variation of the sieve throughput rate before and after jaw crushing.

**Figure 9:**
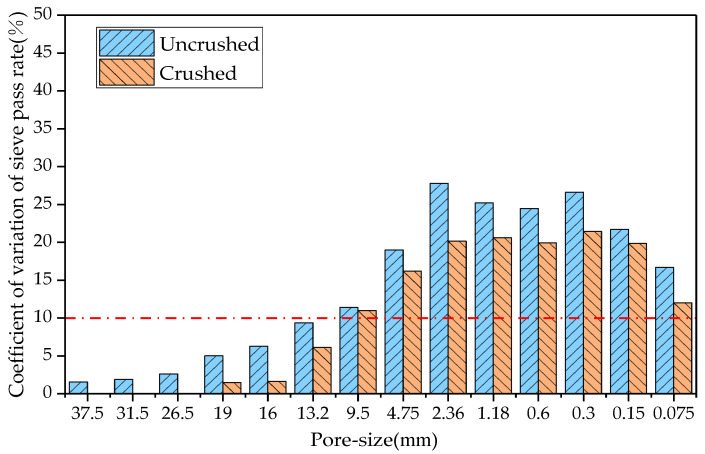
Comparison of the coefficient of variation of the sieve pass rate before and after double roller crushing.

This study determined the RAP material gradation before and after processing of the four crushing methods, compared the coefficient of variation of each sieve passing rate before and after crushing, and the results are shown in Figure 6, Figure 7, Figure 8 and Figure 9.

In probability theory and statistics, a coefficient of variation of less than 10% indicates that the dataset is stable. As can be seen from Figure 6, the coefficients of variation of the sieve passages from 0.3 to 2.36 mm after hammer crushing were 12.1%, 13.4%, 12.8%, and 10.7%, respectively, and the coefficients of variation of the sieve passages of the remaining grain sizes were less than 10%. From Figure 7, the coefficients of variation of the sieve passages from 0.3 to 4.75 mm after small mesh hammer crushing were 13.0%, 13.2%, 10.7%, 10.2%, and 11.0%, respectively, and the coefficients of variation of the sieve passages of the rest of the grain sizes of the RAP were less than 10%. As can be seen from Figure 8, the coefficient of variation of the sieve pass rate of the RAP fine aggregate and 9.5 mm aggregate after jaw crushing treatment was greater than 10%, and the coefficient of variation of the sieve passing rate of the rest of the grain sizes of RAP was lower than 10%. The results in Figure 9 show that double roller crushing reduced the coefficient of variation of the sieve passing rate for aggregates larger than 13.2 mm to less than 10%. From the test results, it can be concluded that small mesh hammer crushing did not reduce the variability of the 4.75 mm aggregate gradation compared with hammer crushing, jaw crushing and double roller crushing had a significant effect on the reduction in the variability of RAP gradation above 13.2 mm, and the variability of the less than 13.2 mm RAP material was still larger after jaw crushing and double roller crushing [26].

**Figure 10:**
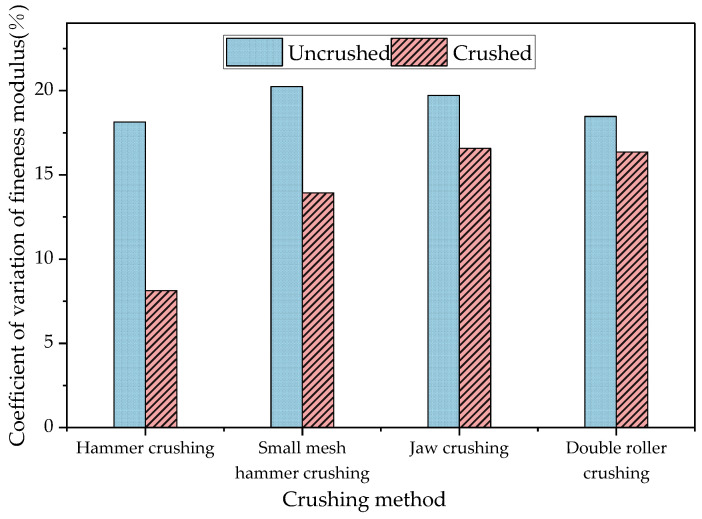
Law of the effect of crushing on the coefficient of variation of the fineness modulus.

In order to analyse the effect of different crushing methods on the variability of the degree of coarseness and fineness of the RAP material, the coefficient of variation of the fineness modulus was used to evaluate the variability of the degree of coarseness and fineness, and the coefficients of variation of the fineness modulus of the RAP material before and after the treatment of the four crushing methods were compared and analysed. The results are shown in Figure 10.

As can be seen from Figure 10, the coefficients of variation of the fineness modulus after treatment by the different crushing methods were all lower than those before crushing, indicating that the four crushing methods could improve the stability of the RAP material gradation and reduce the variability in the degree of coarseness and fineness of the RAP material. This was due to the presence of a large number of agglomerates in the original RAP material, resulting in the aggregate gradation deviating from the original pavement structural ratio with a high degree of discrete aggregate coarseness and fineness. After pretreatment of the agglomerate surface asphalt and fine aggregates, dust and mud and coarse aggregate detachment, an increase in small-sized aggregates and the overall grading refinement, the aggregate asphalt content was reduced so that the RAP material returned to the state of the natural aggregates. Therefore, the overall coarseness and fineness variability of the RAP material was reduced after crushing. The coefficient of variation of the fineness modulus was reduced by 55.18%, 31.18%, 15.97%, and 11.48% after hammer crushing, small mesh hammer crushing, jaw crushing, and double roller crushing, respectively, which indicates that hammer crushing had the strongest ability to reduce the variability of the degree of coarseness and fineness, and the degree of coarseness and fineness basically reached the standard of the original pavement structure after hammer crushing [27].

### 3.2. Effect of Crushing Process on the Dissociation of RAP Material Agglomerates

**Figure 11:**
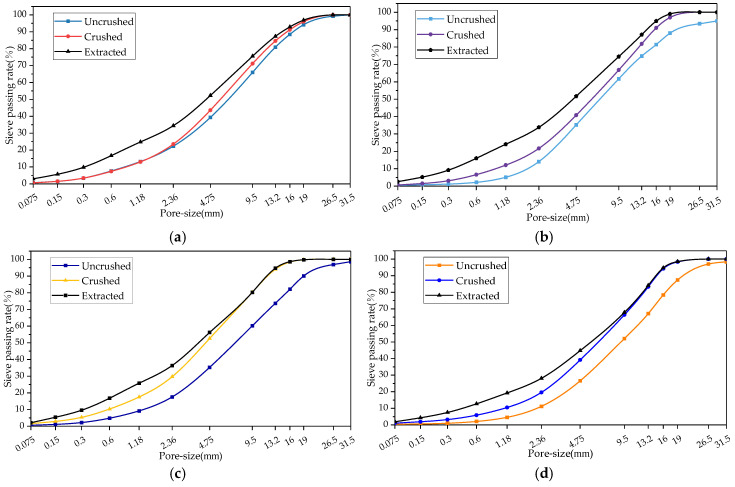
Extraction sieving test results. (**a**) Double roller crushing, (**b**) jaw crushing, (**c**) small mesh hammer crushing, and (**d**) hammer crushing.

**Figure 12:**
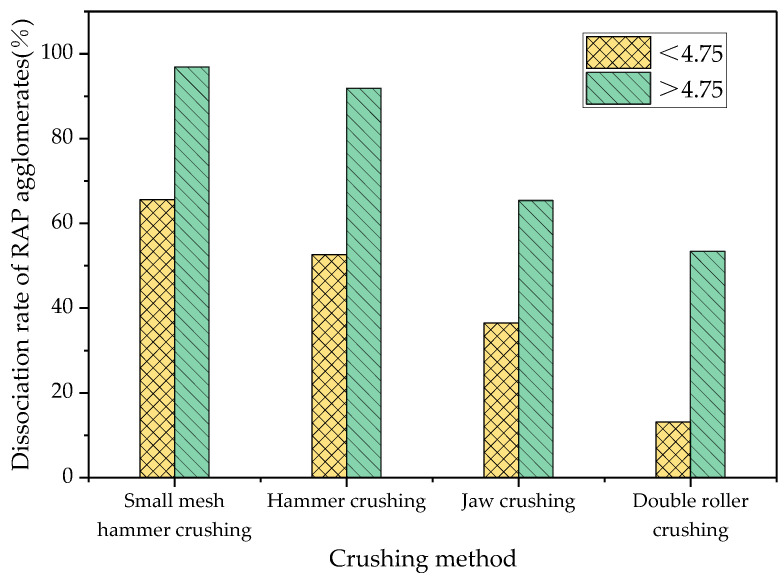
Dissociation rate of the RAP agglomerates after treatment with different crushing methods.

The gradation before and after crushing and before and after extraction were determined using the extraction sieve test, and the degree of dispersion of the clusters was analysed by the agglomeration dissociation rate. The results are shown in Figure 11 and Figure 12.

As can be seen from Figure 11a, the post-extraction gradation curve was above the pumping advance, indicating that agglomeration still existed in the RAP after double-roller shaft crushing. The deviation of grading curves before and after extraction in the range of 0.075~4.75 mm was greater than that in the range of 4.75~31.5 mm, which indicates that the effect of double roller crushing on the agglomeration of coarse aggregates was more significant. It can also be seen from Figure 11a that the 26.5 mm and 31.5 mm sieve passages were the same, which indicates that oil and rock separation was achieved for these two grades of aggregates after double-roller crushing.

From Figure 11b, it can be seen that jaw crushing had little effect on the dissociation of the RAP agglomerates. Except for 26.5 mm and 31.5 mm, where agglomeration did not exist, the rest of the aggregates were still agglomerated. It can also be seen from Figure 11b that the distance between the gradation curves before and after extraction was smaller for the coarse aggregates compared with the fine aggregates, suggesting that jaw crushing had a greater effect on the agglomerate dissociation of the >4.75 mm RAP material than for the fine aggregates.

From Figure 11c,d, it can be seen that the gradation curves after hammer crushing and before and after the extraction of aggregates larger than 4.75 mm basically coincided with each other. This shows that after pretreatment, the coarse aggregate basically achieved oil and stone separation, and the less than 4.75 mm aggregates still existed in an agglomeration phenomenon. It can also be seen from Figure 11c,d that the post-extraction gradation curves deviated less from the pre-extraction distance with the use of small mesh crushing than without, indicating that the RAP was less agglomerated with the use of small mesh than without.

As can be seen in Figure 12, the dissociation rate of the coarse and fine aggregate agglomerates before and after extraction by small mesh hammer crushing was greater than that of the other three crushing modes. The size of the agglomerate dissociation rate of the four crushing methods was as follows: small mesh hammer crushing > hammer crushing > jaw crushing > double roller crushing. The dissociation rate of coarse aggregate agglomerates was greater than that of the fine aggregates for all four crushing methods, which suggests that fine processing has a greater effect on coarse aggregate agglomerates than fine aggregates [28].

### 3.3. Effect of Crushing Process on the Residual Mass Ratio of RAP Materials of Various Particle Sizes

In order to analyse the effect of hammer crushing on the degree of agglomeration of RAP materials of various particle sizes, the changes in the degree of agglomeration of RAP before and after pretreatment were analysed by determining the mass ratio of RAP residue in each grade. The residual mass ratio was expressed as n, where a larger n indicates a lower degree of agglomeration of this grade of RAP material, as shown in Equation (9):

(9) $$n=\frac{m_{1}}{m_{2}}\times 100\%$$

where n is the residual mass ratio of RAP material in the grade, %; $m_{1}$ is the mass of aggregate remaining in the grade after extraction, g; $m_{2}$ is the total mass of RAP material involved in the extraction, g.

**Figure 13:**
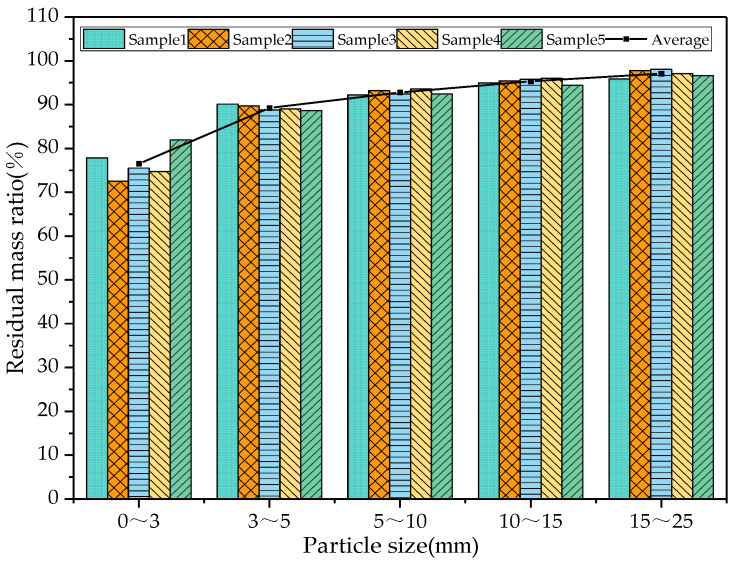
Residual mass ratio of each grade of RAP after hammer crushing.

As can be seen from Figure 13, the residual mass ratio of each grade of RAP material after hammer crushing was greater than 70%, indicating that hammer crushing had an effect on the agglomeration of the RAP material of all grain sizes. The residual mass ratios of the RAP material in the three grain size intervals of 5~10 mm, 10~15 mm, and 15~25 mm reached more than 90%, which indicates that the coarse aggregate basically realised oil–rock separation, but there were still more agglomerates in the fine aggregate, and the crushing had the least effect on the 0~3 mm RAP agglomerates [29].

### 3.4. Analysis of the Mechanical Properties of RAP-Doped Cement-Stabilised Aggregates

#### 3.4.1. Unconfined Compressive Strength

**Figure 14:**
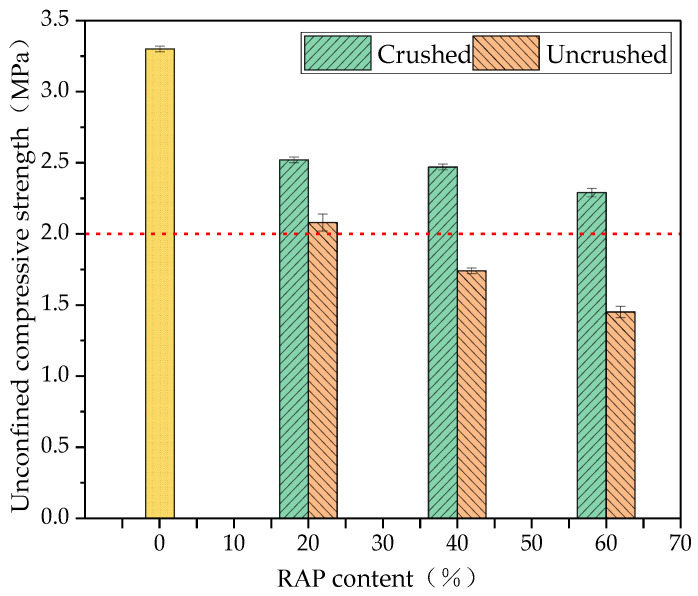
Results of the 7d unconfined compressive strength of cement-stabilised aggregates with crushed and uncrushed RAP.

According to the test results, small mesh hammer crushing dispersed agglomerates with the best effect. However, the small mesh hammer crushing caused the most serious crushing damage to the RAP material, resulting in a shortage of aggregates above 20 mm, so the RAP material was collected after hammer crushing to prepare the mix specimens. The unconfined compressive strength of the cement-stabilised aggregates with 0, 20, 40, and 60% crushed RAP was determined and compared with the cement-stabilised aggregates with uncrushed RAP. The results of the tests are shown in Figure 14.

As can be seen from Figure 14, the 7d unconfined compressive strength of the crushed cement-stabilised recycled mix was higher compared with the uncrushed RAP cement-stabilised aggregates, and the strengths of both the uncrushed and crushed RAP cement-stabilised aggregates were lower than that of the cement-stabilised aggregates when the RAP dosage was zero. The reason for this is that the RAP still contains a small amount of bitumen after the refined processing treatment, which reduces the hydration reaction and is not conducive to the formation of strength due to the contact between the cement paste and the surface of the RAP aggregate. Crushing breaks down the pseudo-grain size in RAP and strips the aged asphalt adhering to the stone surface, improving the mechanical properties of the aggregates, so that the crushed RAP aggregates are more tightly bonded to the cement mortar than the uncrushed, leading to a higher load-bearing capacity of cement-stabilised finely-separated RAP compared with the cement-stabilised aggregates mixed with the original state RAP. With the increase in RAP dosage, the compressive strength of both uncrushed and crushed RAP cement-stabilised crushed stone showed a gradual decrease, and the strength of the crushed cement-stabilised crushed stone decreased by 1.98% and 2.16%, the strength of the uncrushed RAP cement-stabilised crushed stone decreased by 16.34% and 15.38% with the increase in RAP dosage from 20% to 60%, and the strength of the uncrushed RAP cement-stabilised crushed stone decreased by 16.34% and 15.38%, respectively. This indicates that the effect of mixing the original state RAP material to reduce the compressive strength of the cement-stabilised recycled mix is more significant than that after crushing, which is because the grade variability, asphalt content, and agglomerate content of the RAP material after fine processing are reduced, and the nature of the aggregate is more stable than that of the uncrushed RAP. Therefore, the incorporation of crushed RAP had less effect on the performance of the cement-stabilised recycled mixes than that of the uncrushed [30].

As can be seen from Figure 14, the strength of both the uncrushed and crushed RAP cement-stabilised aggregates was lower than that of the unadulterated RAP cement-stabilised aggregates. According to the specification requirements of highway base construction, the 7d unconfined compressive strength of the base of secondary roads and the following highways should reach more than 2 MPa. The strength of the crushed RAP cement-stabilised gravel met the specification requirements, while the strength of 20% RAP dosed in uncrushed RAP cement-stabilised gravel was more than 2 MPa, and the strength of 40% and 60% RAP dosed was less than 2 MPa, which indicates that fine separation can improve the dosage of RAP.

#### 3.4.2. Splitting Strength

**Figure 15:**
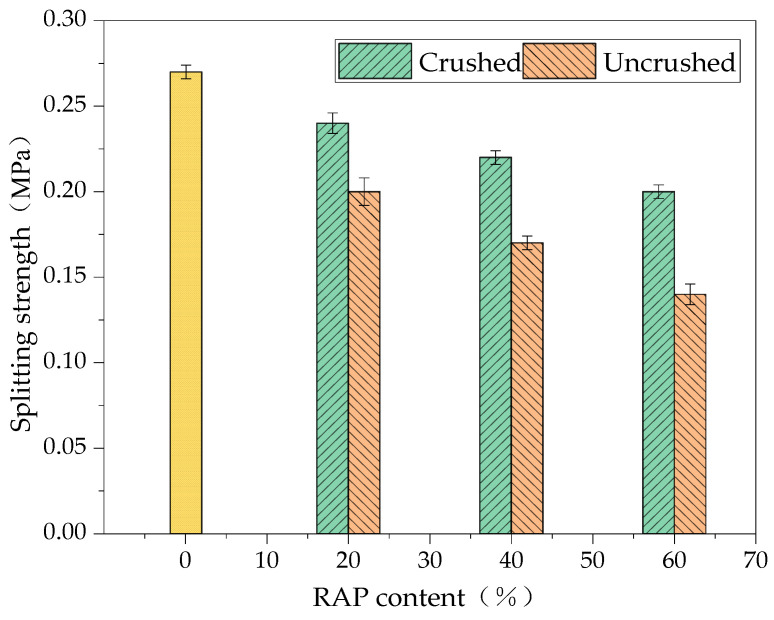
The 7d splitting strength results of the cement-stabilised aggregates with crushed and uncrushed RAP.

The results of the splitting strength test of the RAP-added cement-stabilised aggregates are shown in Figure 15.

As can be seen from Figure 15, the highest splitting strength of the mixture was 0.27 MPa when the RAP dosage was 0. The splitting strength of the uncrushed and crushed RAP cement-stabilised gravel was lower than that of the unadulterated RAP cement-stabilised gravel, and with the increase in the RAP dosage, the splitting strength of the recycled mixture showed a gradual decrease. This indicates that the incorporation of RAP material reduces the cement-stabilised RAP splitting strength. The degree of agglomeration of the crushed RAP material and the low asphalt content caused the splitting strength of the crushed RAP cement-stabilised aggregates to be higher than that of the uncrushed RAP cement-stabilised aggregates. The increase in RAP doping from 20 to 60% after crushing decreased the splitting strength by 8.33% and 8.02%, while the increase in RAP doping from 20 to 60% without crushing decreased the splitting strength by 10.93% and 11.06%. This is because the number of agglomerates of crushed RAP material was less than that of the uncrushed RAP material, and the gradation variability was less than that of the uncrushed RAP material, leading to the strength change of the crushed RAP cement-stabilised aggregates being less than that of the uncrushed RAP cement-stabilised aggregates by increasing the dosage of RAP material [31].

#### 3.4.3. Compressive Modulus of Resilience

**Figure 16:**
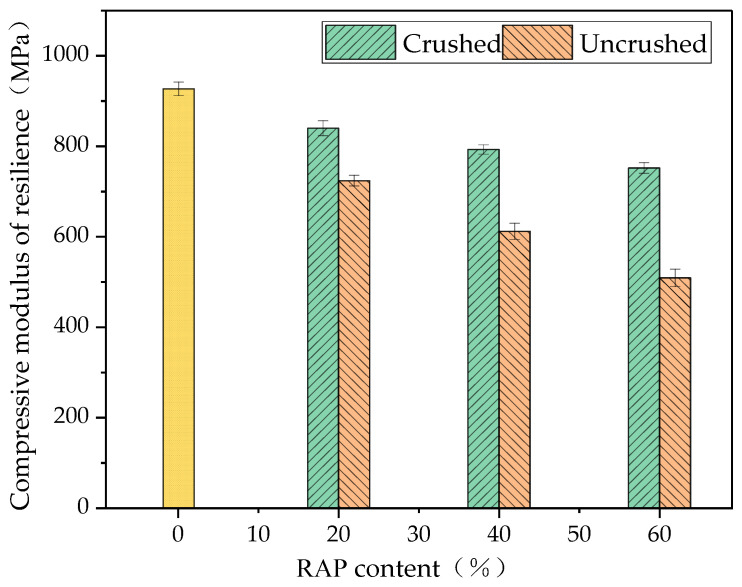
The 7d compressive resilient modulus results of the cement-stabilised aggregates with crushed and uncrushed RAP.

The results of the compressive resilient modulus test of the RAP-doped cement-stabilised aggregates are shown in Figure 16.

As can be seen from Figure 16, the compressive resilience modulus of both the uncrushed and crushed RAP cement-stabilised aggregates was lower than that of the uncrushed RAP cement-stabilised aggregates. This suggests that the aged asphalt adhering to the surface of the RAP material aggregate is flexible, resulting in the cement-stabilised gravel composed of the new aggregate and the RAP material being less stiff than the unadulterated RAP cement-stabilised gravel. The higher the RAP dosage, the smaller the composite stiffness of the regenerated mix, resulting in a gradual decrease in the compressive resilience modulus of the RAP-doped cement-stabilised aggregates with the increase in RAP dosage. The stiffness of the RAP cement-stabilised aggregates with crushing was greater than that of the RAP cement-stabilised aggregates without crushing due to the decomposition of a large number of agglomerates by the crushing process, leading to a reduction in the asphalt content of the RAP material [32].

### 3.5. Micro-Morphological Analysis of the RAP-Doped Cement-Stabilised Aggregates

**Figure 17:**
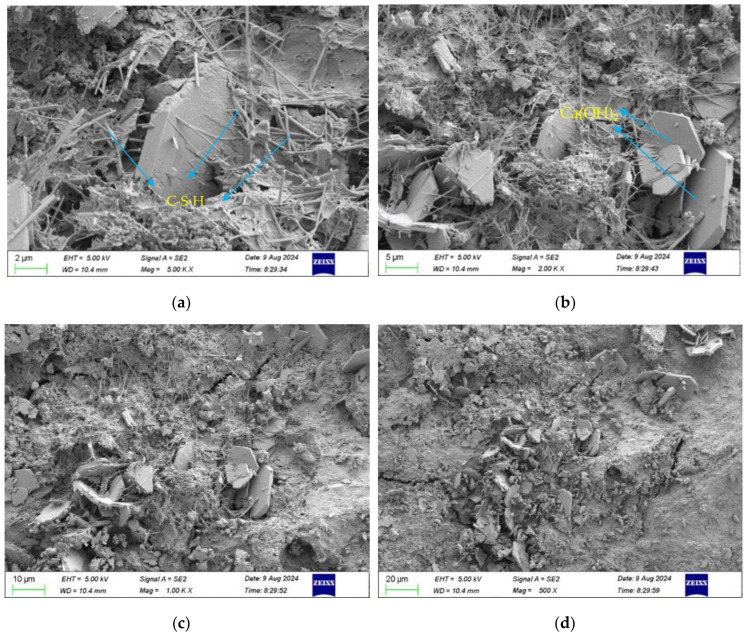
Microscopic morphology of the unadulterated RAP cement-stabilised aggregates. (**a**) 5000× scan. (**b**) 2000× scan. (**c**) 1000× scan. (**d**) 500× scan.

**Figure 18:**
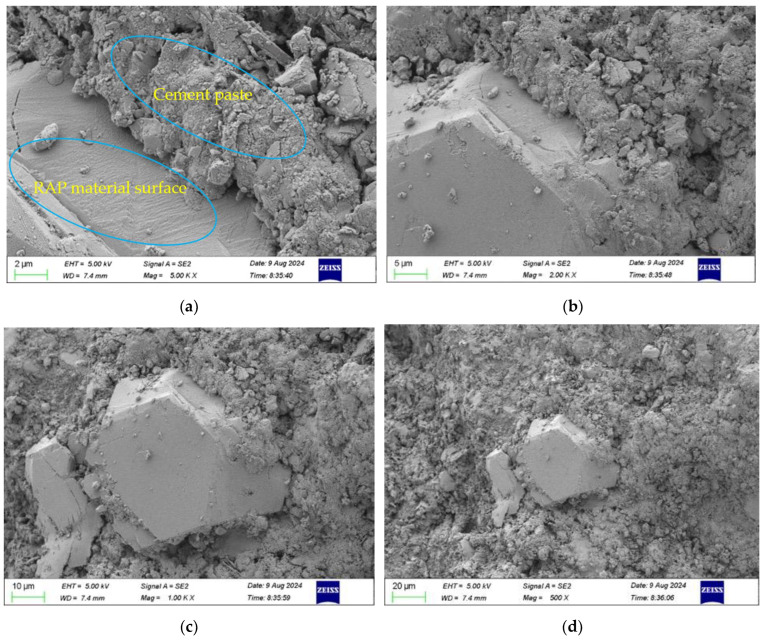
Micro-morphology of the RAP-added cement-stabilised aggregates. (**a**) 5000× scan. (**b**) 2000× scan. (**c**) 1000× scan. (**d**) 500× scan.

Samples of cement-stabilised aggregates without RAP and with 60% RAP admixture were prepared, and the microscopic morphology of both samples is shown in Figure 17 and Figure 18. Tricalcium silicate and dicalcium silicate in cement will react with water to produce calcium silicate hydrate(C-S-H) and calcium hydroxide. As can be seen in Figure 17, when the magnification was 5000 times, it could be observed that the mixture produced a large number of reticulated C-S-H gels and a small number of plate-like Ca(OH)_2_ crystals. Electron microscope images at 2000× magnification showed that C-S-H, calomel, and Ca(OH)_2_ were embedded in each other to form a skeleton, and the contact between the cement paste and the aggregate surface formed a dense structure. From Figure 17c,d, it can be seen that a large amount of hydration products was attached to the surface of the aggregate in the samples, the cement paste was cemented to the stone as a whole, and a small number of tiny holes were still present on the surface of the mix. This was due to the shorter age of the specimens for maintenance and the generation of fewer hydration reaction products, resulting in the hydration products not completely filling the skeleton pores [33].

Figure 18 shows the micro-morphology of the RAP cement-stabilised aggregates, and in Figure 18a, it can be seen that there was a discontinuous transition zone between the RAP and cement paste. In Figure 18b, it can be seen that there were obvious pores and gaps in the transition zone between the RAP and cement paste. Compared with the unadulterated RAP cement-stabilised gravel, the interface of cement-stabilised RAP was discontinuous, so the adhesion between the RAP and cement paste was low, and the macroscopic manifestation was that the compressive strength was less than that of the unadulterated RAP cement-stabilised gravel. The results observed using SEM at 1000 and 500 times magnification are shown in Figure 18c,d. Since asphalt does not participate in the hydration reaction, and at the same time is not conducive to the contact between the aggregate and the hydration products, the amount of calcium hydroxide, calcite crystals, and hydrated calcium silicate colloidal hydration products in the mix was less than that of the unadulterated RAP cement-stabilised crushed stone, and the overall structure was more sparse and did not form a solid whole, which led to the low mechanical properties of the cement-stabilised RAP [34].

### 3.6. Relationship Between RAP Material Properties and Mechanical Properties of RAP-Doped Cement-Stabilised Aggregates

**Figure 19:**
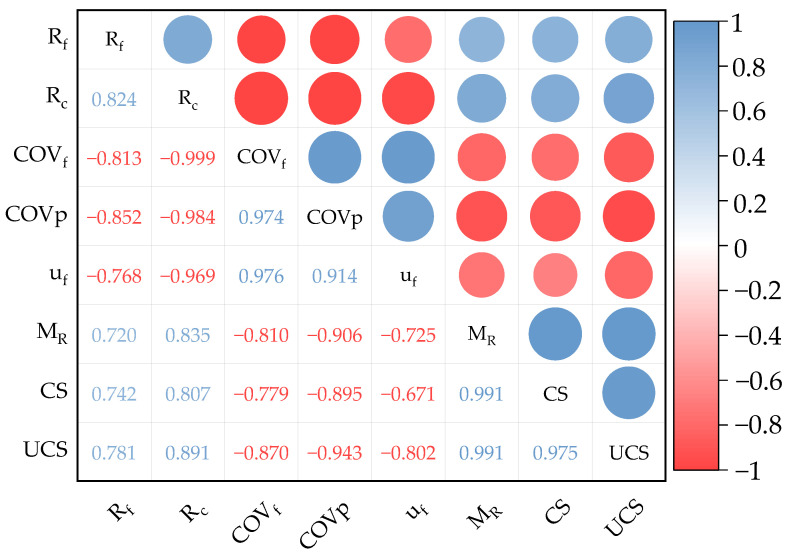
Correlation analysis between the RAP material properties and mechanical properties of the RAP-doped cement-stabilised aggregates.

In order to explore the relationship between the RAP material properties and mix mechanical properties more deeply, Pearson correlation analyses were carried out between the coarse aggregate agglomerate dissociation rate ($R_{c}$), fine aggregate agglomerate dissociation rate ($R_{f}$), mean coefficient of variation of sieve passage rate ($COV_{p}$), coefficient of variation of fineness modulus ($COV_{f}$), and fineness modulus ($u_{f}$) of the RAP materials and the mix compressive strength (UCS), splitting strength (CS), and compressive modulus of elasticity of return ($M_{R}$). The correlation coefficient R^2^ describes the degree of linear correlation between two variables and its calculation is shown in Figure 19.

As can be seen from Figure 19, the absolute values of the correlation coefficients between the compressive strength of the RAP-added cement-stabilised aggregates, the splitting strength, compressive modulus of elasticity, and the material properties of the RAP materials were all higher than 0.6. This indicates that there is a strong correlation between the RAP material properties and the mechanical properties of the recycled mixes. The correlation coefficients between the mechanical properties of the mixes and the dissociation rates of the coarse and fine RAP material agglomerates were all greater than 0.7, and the effect of the coarse aggregate agglomerates on the mechanical properties of the mixes was higher than that of the fine aggregates. This was due to the fact that crushing reduced the number of agglomerates in the coarse RAP material more significantly than in the fine RAP material, and the degree of agglomeration in the RAP material smaller than 4.75 mm remained high after processing.

It can also be seen from Figure 19 that the mean values of the coefficient of variation of the fineness modulus and the coefficient of variation of the sieve passage rate correlated well with the mechanical properties of the mixes, with an average R^2^ of −0.819 and −0.915, respectively, suggesting that the variability of the RAP material gradation led to a reduction in the mechanical properties of the mixes. The fineness modulus had the lowest effect on the mechanical properties of the mix. The coarse aggregate agglomerate dissociation rate had a very high correlation with the coefficient of variation of the fineness modulus and the coefficient of variation of the sieve passage rate, with R^2^ reaching −0.999 and −0.984, respectively. This indicates that the grading variability is mainly caused by RAP coarse aggregate agglomerates, and the phenomenon of RAP material agglomeration leads to changes in the skeleton of the mixture, which results in large fluctuations in the mechanical properties of cement-stabilised aggregates with high RAP dosage, making it difficult to meet the technical requirements.

## 4. Conclusions

The objective of this study was to investigate the impact of the crushing process on the grading and agglomeration characteristics of the RAP materials and to ascertain the mechanical properties of the RAP-doped cement-stabilised aggregates. The microscopic morphology of cement-stabilised aggregates with crushed and uncrushed RAP was observed, and the relationship between the characteristics of the RAP materials and the mechanical properties of the RAP-doped water-stabilised base was analysed. The main conclusions are as follows:

(1) Hammer crushing improved the full-size RAP material sieve throughput, and jaw and double roller shaft crushing significantly affected the coarse aggregate gradation. The highest degree of RAP material refinement was achieved after small mesh hammer crushing, with a reduction in fineness modulus of 13.55%. Hammer crushing reduced the RAP material grade variability best.
(2) The small mesh hammer crushing after agglomeration dissociation rate was the highest, and the crushing and decomposition of the coarse aggregate agglomeration effect was better than the fine aggregates. After hammer crushing, the RAP coarse aggregate residual mass ratio reached more than 90%, and the fine aggregate bitumen content remained high.
(3) Hammer crushing improved the compressive and splitting strengths of the RAP-doped cement-stabilised aggregates compared with uncrushed crushing, increased the stiffness of the mix, and increased the RAP dosage to 60%. The hydration reaction product of the RAP cement-stabilised gravel was less than that of the RAP cement-stabilised gravel after crushing, the combination of RAP material and cement paste was not close, and the adhesion was low.
(4) There was a high correlation between the RAP material gradation variability, agglomeration condition, and the mechanical properties of the RAP-doped cement-stabilised aggregates, with the fineness modulus having the lowest effect on the mechanical properties of the recycled mixes. The RAP coarse aggregate agglomerate dissociation rate correlated with the coefficient of variation of the sieve passage rate and the coefficient of variation of the fineness modulus up to −0.984 and −0.999, respectively.

## Figures and Tables

**Table 1:** RAP material and natural aggregate mud content.

Mud Content	Particle Size				Technical Requirement	Test Method
Particle size range (mm)	5~10	10~15	15~20	20~25	≤1.2	T 0310
RAP (%)	0.91	0.75	0.72	0.72		
Limestone (%)	0.42	0.11	0.28	0.31		

**Table 2:** Needle-flake particle content of RAP material and natural aggregate.

Needle-Flake Particle Content	Particle Size				Technical Requirement	Test Method
Particle size range (mm)	5~10	10~15	15~20	20~25	≤18	T 0312
RAP (%)	2.94	4.01	7.69	9.05		
Limestone (%)	5.35	5.93	10.66	11.41		

**Table 3:** RAP material and natural aggregate crushing value.

Crushing Value	Test Number			Average Value (%)	Technical Requirement	Test Method
	1	2	3			
RAP (%)	20.8	19.4	20.3	20.2	≤22	T 0316
Limestone (%)	16.6	16.9	18.1	17.2		

**Table 4:** Cement properties.

Test Item		Technical Requirement	Result
Fineness (%)		<10%	7.2
Solidification time (min)	Initial setting	≥180 min	295
	Final setting	≥360 min and ≤600 min	407
Break off strength (MPa)	3 d	≥4.0	5.8
	28 d	≥6.5	7.6
Compressive strength (MPa)	3 d	≥17	22.4
	28 d	≥42.5	47.3

## Data Availability

The original contributions presented in this study are included in the article. Further inquiries can be directed to the corresponding authors.
